# A Systematic Review of Attentional Bias in Problem Gambling

**DOI:** 10.1007/s10899-023-10260-9

**Published:** 2023-11-09

**Authors:** Zoe Farr, Niall M. Broomfield, Kenny R. Coventry

**Affiliations:** 1 Department of Clinical Psychology, Norwich Medical School, University of East Anglia, Norwich, UK; 2 School of Psychology, University of East Anglia, Norwich, UK

**Keywords:** Attentional bias, Problem gambling, Gambling disorder, Systematic review

## Abstract

A large body of previous research has provided support for the role of attentional bias as a maintaining factor in addiction. This systematic review aimed to investigate the extent and nature of attentional bias as a phenomenon which exists within problem gamblers**.** Studies were identified through searches of three databases (MedLine, PSYCHINFO, and Web of Science) and examination of the reference lists of the final studies meeting criteria for inclusion. The scope of the review included empirical studies making experimental comparisons of problem gamblers and non-problem gamblers across a range of attentional paradigms. A comparison of effect sizes was conducted across studies comparing problem to non-problem gamblers within and between attention paradigms. Twenty-two studies were reviewed systematically across ten experimental paradigms. Attentional bias was demonstrated in 16 of the 22 studies, with attentional bias effects varying across paradigms. Quality assessment revealed two main limitations across studies: lack of a priori power analysis, and failure to control for gambling frequency as a possible confounding variable. Findings support the role of attentional bias as a potential maintaining factor in problem gambling behaviour, in line with evidence for substance addiction. Recommendations for future studies are outlined alongside a discussion of clinical implications.

## Introduction

Gambling disorder is defined as ‘persistent and recurrent problematic gambling behaviour leading to clinically significant impairment or distress’ (American Psychiatric Association, 2013), and is thought to affect around 0.5% of British adults (Public Health England, 2021). The Diagnostic and Statistical Manual of Mental Disorders, Fifth Edition (DSM-5; American Psychiatric Association, 2013) introduced Gambling disorder as the first and only behavioural addiction, representing a shift from the previous understanding of ‘pathological gambling’ as an impulse control disorder in response to the increasing evidence for etiological parallels with substance use disorders (Reilly & Smith, 2013). Similarities between the disorders include behavioural manifestations (e.g. inability to stop, progression and patterns of escalation), shared comorbidities, genetic vulnerabilities, and responses to specific pharmacologic treatments (Pallanti et al., 2021). Traits such as impulsivity and compulsivity have also been associated with both problem gambling and substance use disorders, and similar areas of dysfunction have been identified in the brain (Leeman & Potenza, 2012).

In recent years attentional bias has become a significant focus in addiction research, with a burgeoning evidence base for the increased salience of substance-related stimuli in substance users compared to controls (Marks et al., 2014). In line with the numerous parallels between problem gambling and substance use disorders, theories of attentional bias related to substance misuse have been increasingly applied to problem gambling. For example, Brevers et al. (2011a) applied the incentive-sensitisation theory (Robinson & Berridge, 1993) to problem gambling, describing how sensitisation of the brain’s meso-limbic and meso-cortical dopamine systems generate incentive motivation for gambling behaviours, producing attentional bias as a means of reward-seeking. Similarly, Grant and Bowling (2015) extended Tiffany’s (1990) cognitive model of drug use to problem gambling, whereby continued participation in gambling produces automatic unconscious bias towards gambling-related stimuli. Cox et al. (2016) also highlight the application of the ‘theory of current concerns’ (Klinger & Cox, 2004) to the phenomena of attentional bias in addiction, noting that greater concern (motivational goal-striving) about an addictive substances or behaviour would translate in greater attentional bias for addiction related stimuli.

An empirical distinction has been drawn between attentional bias at the point of attention orientation (facilitated attention) contrasted with bias in maintenance of attention (difficulty with disengagement). This differentiation is typically accomplished via manipulation of the length of stimulus presentation, where presentations of ≤ 200 ms measure a rapid automatic orienting of attention, and more sustained presentations of ≥ 500 ms reflect a sustained maintenance of attention (Fernández-Calderón et al., 2021).

Attaining a comprehensive understanding of the role of attentional bias in problem gambling is crucial for enriching comprehension of the phenomenon's underlying mechanisms, potential contribution to the maintenance of problem gambling behaviour and guiding the development of effective psychological treatment approaches. Furthermore, distinguishing between attentional bias at the stage of orientation and maintenance of attention is fundamental in advancing our understanding of the phenomenon while also informing the development of clinical interventions. Specifically, understanding whether attentional bias occurs rapidly at initial orientation or presents as a delay in disengaging from gambling stimuli could guide the development of appropriately targeted attentional bias modification programs which reflect any potential differences in the degree of conscious control (Cicaerelli et al., 2019; see Field & Cox, 2008 for further discussion).

### Objectives

The main research question for this systematic review is ‘What is the empirical evidence on attentional bias in problem and pathological gamblers?’.

The objectives of the current review are fourfold. It seeks to outline the magnitude of any observed attentional bias effects, establish the quality of included studies, and consider the processes of initial orientation and maintenance of attention.

It also aims to provide recommendations for future research and discuss the clinical implications of the empirical evidence. A review was previously conducted by Hønsi et al. (2013), however a number of relevant studies have been published since this time, and as such the current paper allows examination of a larger, more robust evidence base.

## Method

The protocol for this systematic review was registered on the PROSPERO international prospective register of systematic reviews on 23rd May 2022 (registration number CRD42022306333) and adheres to the Preferred Reporting Items for Systematic Reviews and Meta-Analyses (PRISMA) guidelines (Moher et al., 2009).

### Search Strategy

Searches were conducted across MedLine, PsycInfo, and Web of Science databases, in August 2022. The search strategy included the following terms: (gambling OR gambler OR gamblers OR gambling OR gambl*) AND (attention OR attentional OR attention*) AND bias. The reference lists of the final studies which met criteria for inclusion were also reviewed.

### Eligibility Criteria

The review includes empirical studies which make experimental comparisons of problem gamblers and a control group (non-problem gamblers or non-gamblers). Only studies written in English and published in peer-reviewed journals were considered for inclusion. Intervention studies (e.g. RCT’s) were excluded from this review.

### Study Screening and Quality Assessment

**Fig. 1:**
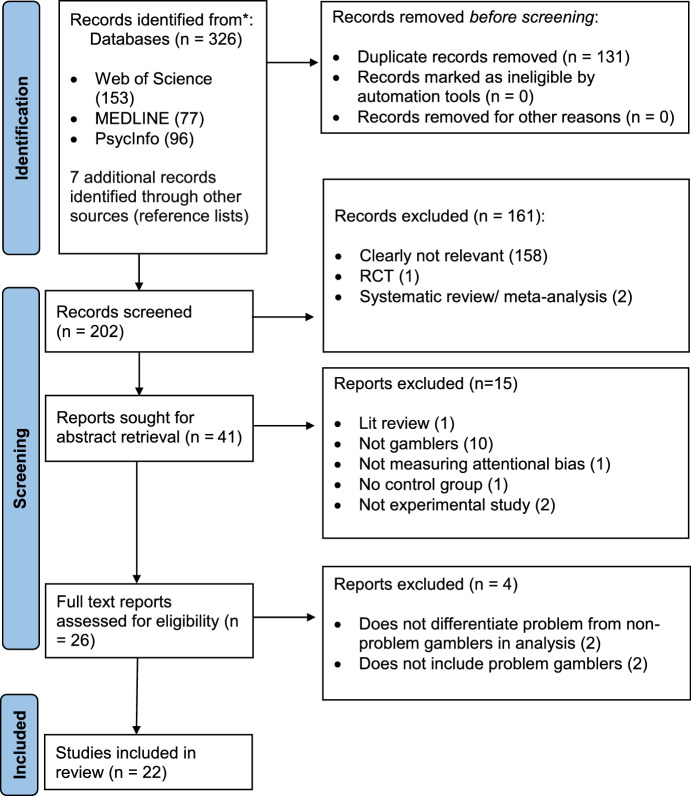
PRISMA flow diagram of the selection process

In line with PRISMA guidelines (Moher et al., 2009) (see Fig. 1), the selection process was completed by two reviewers, both experts on gambling addiction (to reduce the likelihood of rejecting relevant studies). The second reviewer considered twenty percent of the studies screened by the primary reviewer at the first two stages, and fifty percent at the final stage. Out of the 202 titles screened, the second reviewer screened 40 achieving an agreement rate of 100%. Out of the 41 abstracts screened, the second reviewer screened 8 with a 100% agreement rate. Finally, of the 26 full text articles screened, the second reviewer screened 13 with a 76.9% agreement rate. Reviewers jointly examined inclusion and exclusion criteria for each article where there was a discrepancy to reach a final consensus.

To appraise the quality of included studies, a checklist of eleven questions was formulated based on existing quality assessment checklists (see Appendix for checklist and rationale), specifically the Appraisal tool for Cross-Sectional Studies (AXIS) (Downes et al., 2016), which address the quality of reporting, study design quality, and biases. The most relevant seven questions from the AXIS were selected jointly between the two reviewers, a further two questions were adapted from Critical Appraisal Skills Programme (CASP) checklists (2018, 2020) and one question was adapted from the Scottish Intercollegiate Guidelines Network (SIGN) checklist for case–control studies (2012). One additional question pertaining to the inclusion of control conditions was generated by the reviewers as an assessment of internal validity (Torday & Baluška, 2019). A third reviewer (expert on addiction) undertook quality assessment for 11 of the 22 studies (50%) with a 74% agreement rate, following which discrepancies were discussed to reach a final consensus. Each study received an overall percentage rating based on the proportion of checklist criteria met (M = 80.56%, SD = 7.05). Quality assessment for each study is presented in Table 1, with studies grouped by paradigm and ordered chronologically.

**Table 1:** Results of quality assessment

Study	Assessment quality criteria											
	1	2	3	4	5	6	7	8	9	10	11	Total (%)
*Stroop*												
McCusker and Gettings (1997)	+	−	−	−	+	−	+	+	+	+	+	63.6
Atkins and Sharpe (2003)	+	−	+	−	+	+	+	+	+	+	+	81.8
Boyer and Dickerson (2003)	+	−	+	+	+	+	+	+	+	+	+	90.9
Molde et al. (2010)	+	+	+	−	+	+	+	+	+	+	+	90.9
Cutter (2016)	+	−	+	−	+	+	+	+	+	+	+	81.8
*Attentional blink*												
Brevers et al. (2011b)	+	−	+	−	+	−	+	+	+	+	+	72.7
Hudson et al. (2016)	+	−	+	−	+	+	+	+	+	−	+	72.7
*Dual task*												
Diskin and Hodgins (1999)	+	−	+	−	−	−	+	+	+	+	+	63.6
Diskin and Hodgins (2001)	+	−	+	−	−	+	+	+	+	+	+	72.7
*Lexical salience*												
Zack and Poulos (2004)	+	−	+	−	+	+	+	+	+	+	+	81.8
Zack and Poulos (2007)	+	−	+	−	+	+	+	+	+	+	+	81.8
*Flicker-induced change blindness*												
Brevers et al. (2011a)	+	−	+	−	+	+	+	+	+	+	+	81.8
*EEG cue reactivity*												
Wölfling et al. (2011)	+	−	+	−	+	+	+	+	+	+	+	81.8
*Approach avoidance*												
Boffo et al (2018)	+	−	+	+	+	+	+	+	+	+	+	90.9
*Posner*												
Ciccarelli et al. (2016a)	+	−	+	−	+	+	+	+	+	+	+	81.8
Ciccarelli et al. (2016b)	+	−	+	−	+	+	+	+	+	+	+	81.8
Ciccarelli et al. (2019)	+	−	+	−	+	+	+	+	+	+	+	81.8
Ciccarelli et al. (2020)	+	−	+	−	+	+	+	+	+	+	+	81.8
*Eye tracking*												
McGrath et al. (2021)	+	−	+	−	+	+	+	+	+	+	+	81.8
Kim et al. (2021)	+	+	+	−	+	+	+	+	+	+	+	81.8
Kim et al. (2022)	+	+	+	−	+	+	+	+	+	+	+	90.9
*Visual Probe*												
Vizcaino et al. (2012)	+	−	+	−	+	+	+	+	+	+	+	81.8

Criteria: (1) Were the aims/objectives of the study clear? (2) Was the sample size justified (e.g. power analyses)? (3) Was membership in a ‘problem gambling’ group established through use of a reputable screening tool (e.g. PGSI/SOGS/DSM-V)? (4) Were the gambling and control group(s) matched for gambling frequency as a confounding variable? (5) Were additional conditions included to offer a comparison to performance in gambling conditions? (6) Were the experimental and control groups sampled from the same population? (7) Was the selection process likely to select subjects/participants that were representative of the target/reference population under investigation? (8) Were the outcome variables measured appropriate to the aims of the study? (9) Is it clear what was used to determined statistical significance and/or precision estimates (e.g. *p*-values, confidence intervals)? (10) Were the basic data adequately described? (11) Were the results presented for all the analyses described in the methods? +  = yes; − = no

### Data Extraction and Analysis

Data were extracted on participant numbers and gender, measurement of problem gambling severity (e.g. Problem Gambling Severity Index [PGSI; Ferris & Wynne, 2001], South Oaks Gambling Screen [SOGS; Lesieur & Blume, 1987]), and study design. Effect sizes (Cohen’s* d*) were calculated for each study to demonstrate the magnitude of any reported effect. Where the relevant data was not available in published papers the authors were contacted to request this. Contact was made in relation to three of the 22 papers, however no responses were received and effect sizes were thus calculated based on available data. Details of the final 22 included studies are outlined in Table 2.

**Table 2:** Summary of studies

Method and study	Participants	Scores on measure of gambling severity (SD)	Measure	Attentional bias	Effect size (Cohen’s d)
*Stroop task*					
McCusker and Gettings (1997)	PG = 15, all males PG's spouses = 15, 0 males HC = 15, 8 males	None	Gambling, drug-related and neutral word stimuli	Attentional bias among PG at orienting of attention for gambling-related stimuli; PG significantly slower than HC/spouses to respond to gambling stimuli	PG/spouses*: d* = 1.43^a^ PG/HC*: d* = 2.08^a^
Atkins and Sharpe (2003)	PG = 12, 8 males HFG = 12, 8 males LFG = 12, 8 males	SOGS: PG = 10.92 (1.50) HFG = 1.17 (0.898) LFG = 0.25 (0.60)	Positive and negative gambling-related, emotional and neutral word stimuli	Significant interaction effect between group and condition. Reverse interference effect: PG responded *more quickly* to positive gambling words in comparison to controls	PG/Controls:* d* = − 0.735^bh^ PG/HFG: *d* = 1.551^a^ PG/LFG: *d* = 1.088^a^
Boyer and Dickerson (2003)	Low control = 30, 13 males High control = 30, 7 males	Scale of gambling choices (SGC) Low control = 23.93, (4.17) High Control = 50.70, (11.11)	Gambling, drug-related and neutral word stimuli	Significant interaction effect between group and condition. Attentional bias for gambling stimuli among low control group at orienting of attention	*d* = 0.517^b^ *d* = 0.189^a^
Molde et al. (2010)	PG = 33, 26 males HC = 22, 16 males	SOGS PG = 11.75 (2.49) HC = 0.59 (1.01)	Win-related and neutral pictorial stimuli, supraliminal and subliminal conditions	Significant interaction effect between group and condition. Attentional bias at orienting of attention among PG for win-related stimuli in both conditions	*d* = 0.633^b^ PG/HC win-related stimuli *d* = 0.668^a^
Cutter (2016)	PG = 10 MPG - 26 LPG - 18 NPG - 6 Total sample: 44 males, 16 females	PGSI PG = ≥ 8 MPG = 3–7 LPG = 1–2 NPG = 0	Gambling related, negative and neutral word stimuli	No significant interaction between group and condition. No attentional bias effect	N/A
*Attentional Blink*					
Brevers et al. (2011b)	PG = 40, 22 males HC = 35, 20 males	SOGS PG = 4.6 (2.71) HC = Not administered	Gambling- related and neutral word stimuli	Significant interaction effect between group, condition, and lag. Attentional bias among PG for gambling-related words at orienting of attention (200 ms)	*d* = 0.585^b^ *d* = 0.532^a^
Hudson et al. (2016)	High risk gamblers - 31, 21 males Low risk gamblers - 26, 14 males	PGSI High-risk = 7.45 (4.26) Low-risk = 1.04 (0.82)	Gambling and non-gambling pictorial stimuli (positive, negative and neutral)	No significant attentional bias effect	N/A
*Dual-task*					
Diskin and Hodgins (1999)	PG = 12, 6 males Occasional gamblers = 11, 4 males	SOGS: PG = 9.8 (3.0) OG = 1.7 (1.4)	Video lottery play while responding to external light	No significant interaction between group and condition (time period). Attentional bias among PG at maintenance of attention	*d* = 1.179^c^
Diskin and Hodgins (2001)	PG = 20, 9 males Occasional gamblers = 22, 10 males	SOGS: PG = 9.8 (3.0) OG = 1.7 (1.4)	As in Diskin and Hodgins, (1999) with inclusion of a baseline measure	Significant interaction between group and condition order. No significant difference between groups on reaction times; no attentional bias effect	*d* = 1.248^b^ *d* = 0.052^d^
*Lexical salience*					
Zack and Poulos (2004)	PG = 10, 7 males PG + D = 6, 4 males D = 8, 5 males HC = 12, 9 males	SOGS PG = 8.4 (3.4) PG + AD = 8.0 (3.3) AD = 0.6 (1.1) HC = 0.2 (0.4)	Gambling-related, alcohol-related, positive, negative and neutral word stimuli AMPH D2 agonist and placebo conditions	No attentional bias in placebo for gambling related stimuli between PG and HC	N/A
Zack and Poulos (2007)	PG = 20, 17 males HC = 18, 14 males	DSM diagnosis, no SOGS/ PGSI score reported	As described in Zack and Poulos (2004) Haloperidol DA D2 antagonist and placebo conditions	No attentional bias in placebo for gambling related stimuli between PG and HC	N/A
*Flicker-induced change blindness*					
Brevers et al. (2011a)	PG = 40, 22 males HC = 35, 20 males	SOGS PG = 4.6 (2.71) HC = 0.0 (0.0)	Flicker task with eye-movement monitoring; gambling-related and neutral pictorial stimuli Flicker task with eye-movement monitoring; gambling-related and neutral pictorial stimuli	Significant interaction effect between groups on change detection latency, proportion of fixation count and fixation length. Difference between means on first eye movement percentages. Attentional bias among PG at orientation and maintenance of attention	Change detection latency: *d* = 0.76^b^ Direction of first eye movement toward gambling pictures: *d* = 1.09^a^ Proportion of fixation count: *d* = 0.577^b^ Fixation length: d = 0.734^b^
*EEG cue-reactivity*					
Wölfling et al. (2011)	PG = 15, 12 males HC = 15, 13 males	SOGS PG = ≥ 5 HC = not reported	Gambling and non-gambling stimulus material (positive, negative and neutral)	Significant interaction effect between group and stimulus category. Attentional bias (LPP’s) among PG at maintenance of attention	*d* = 1.373^b^
*Approach avoidance*					
Boffo et al. (2018)	Moderate/ high risk gamblers = 22, all male Non-PG = 26, all male	PGSI Moderate/ high risk = 5.32 (2.48) Non-PG = 1.08 (0.84)	Gambling and neutral pictorial stimuli	Significant interaction effect between group and stimulus. Attentional bias (approach bias) among PG (moderate- to high-risk gamblers) for gambling stimuli at orientation of attention	Baseline: *d* = 0.639^b^ *d* = 0.38^e^ Follow-up: *d* = 0.745^e^
*Posner*					
Ciccarelli et al. (2016a)	PG = 25, all male Non-PG = 25, all male Abstinent PG = 25, all male	SOGS PG = ≥ 3 Non-PG = ≤ 2 Abstinent PG = DSM diagnosis of GD	Gambling related and neutral pictorial stimuli	Significant interaction between group, validity, and stimulus valence Attentional bias (facilitation bias) among PG for gambling stimuli at orientation of attention (100 ms)	*d* = 1.028^f^
Ciccarelli et al. (2016b)	PG = 54, all male Non-PG = 54, all male	SOGS PG = ≥ 3 Non-PG = ≤ 2	Gambling related and neutral pictorial stimuli	No significant interaction effects between group and valence. Attentional bias (facilitation bias) among PG compared for gambling stimuli at orientation of attention (100 ms)	*d* = 0.865^f^
Ciccarelli et al. (2019)	PG = 33 Non-PG = 54 Total sample: 82 males, 5 females	SOGS-RA PG = ≥ 2 HC = ≤ 1	Gambling related and neutral pictorial stimuli	No significant interaction effects. Attentional bias among PG at maintenance of attention (500 ms)	*d* = 0.701^b^
Ciccarelli et al. (2020)	PG = 28, all male HC = 42, all male	SOGS PG = ≥ 2 HC = ≤ 1	Gambling related and neutral pictorial stimuli	Significant interaction between group and time. Attentional bias among PG at orienting of attention (100 ms)	*d* = 0.701^b^
*Eye tracking*					
McGrath et al. (2021)	No-risk = 38 Low-risk = 24 Moderate/High-risk = 25 Gender of sample not specified	PGSI No risk = 0.0 Low risk = 2.4 Moderate/ High-risk = 6.6	Gambling related and neutral pictorial stimuli	Significant interaction between group and attentional bias scores. Attentional bias among PG (Moderate/High risk group) at maintenance of attention	*d* = 0.78^f^ PG/No-risk: *d* = 1.361^g^ PG/ Low-risk: *d* = 0.638^g^
Kim et al. (2021)	PG EGM players = 25, 13 males Non-PG EGM players = 52, 26 males HC = 60, 28 males	PGSI PG = ≥ 5 Non-PG = 0–4 HC = Not reported	Gambling (EGM) and neutral images	Significant interaction between group and stimulus type. Attentional bias among PG orientation of attention	*d* = 1.329_2_ PG/HC: *d* = 2.55^g^
Kim et al. (2022)	PG = 25 Non-PG = 50 Total sample: 38 males, 37 females	PGSI PG = ≥ 5 Non-PG = 0–4	Gambling (EGM) and neutral images	Attentional bias among PG at orientation of attention	PG/Non-PG: *d* = 1.38^g^
*Visual probe*					
Vizcaino et al. (2012)	PG = 23, 21 males Non-PG = 21, 16 males	SOGS PG = 11.9 (2.7) Non-PG = 1.2 (0.4)	Gambling related and neutral pictorial stimuli	Attentional bias among PG at maintenance of attention	*d* = 1.023^g^

*PG* Problem gamblers, *HC* Healthy controls, *MPG* Moderate problem gambling, *LPG* Low problem gambling, *NPG* Non problem gambling, *HFG* High frequency gamblers, *LFG* Low frequency gamblers, *PG + D* Gambler-drinkers, *D* Drinkers,* EGM* Electronic gaming machine, *GD* Gambling disorder, *SOGS-RA* South Oaks Gambling Screen Revised for Adolescents (Winters et al., 1993).

Cohen’s *d* effect size: small (d = 0.2), medium (d = 0.5), and large (d = 0.8)

^a^Between group performance on gambling stimuli. ^b^Interaction effect. ^c^Between group reaction times. ^d^Between group difference score (baseline vs VLT reaction time). ^e^Between groups gambling approach bias, ^f^Attentional bias for problem gamblers (within group), ^g^ Between group attentional bias for gambling stimuli over neutral stimuli.^h^ Value is calculated from a combination of two control groups, and the reporting of analysis is of poor quality

It was not feasible to conduct a meta-analysis within the current review due to methodological heterogeneity across paradigms. Cochrane advises a minimum of two studies to conduct meta-analysis (Ryan, 2016; cf. McShane & Böckenholt, 2017), and whilst there are 22 studies included with the review, these exist across 10 attentional bias paradigms, with four paradigms including only one study.

## Results

10 measures of attentional bias were used across the 22 included studies (Addiction Stroop, Attentional blink, Dual-task, Lexical salience, Flicker-induced change blindness, EEG cue reactivity, Approach avoidance, Posner, Eye-tracking, and Visual probe). The studies under each paradigm are examined in turn.

### Addiction Stroop Task

The addiction Stroop task measures the interference of addiction-related stimuli compared to neutral stimuli, where attentional bias is gauged through comparing colour-naming reaction times between the word categories (Field et al., 2009). The cognitive interference observed in the addiction Stroop task is largely considered to reflect attentional bias at the initial orienting of attention (McCusker & Gettings, 1997), however Field et al. (2009) reason that the addiction Stroop task should be considered as a variant of the emotional Stroop task, highlighting carry-over effects in the relevant literature indicate a slow disengagement of attention.

McCusker and Gettings (1997) employed a Stroop task with gambling, neutral, and drug-related words with 15 male recruits from Gamblers Anonymous. Controls were spouses of the gamblers and 15 additional controls comprised of eight male and seven female staff and students from a university. No screening tools were utilised to establish gambling psychopathology and group allocation was reliant on self-reports of gambling behaviour, with the parameters of group membership not clearly defined in the research paper. Gamblers demonstrated a significant increase in reaction times for gambling-related words as compared to controls demonstrating greater cognitive interference (*d* = 2.08), and a further post-hoc analysis revealed an additional effect of gambling type specificity, with racing gamblers and fruit machine players demonstrating greater attentional bias to gambling stimuli of individual relevance, though the sample size was limited (n = 11). Moreover, the analyses reported no significant interaction effect between groups and stimulus type, indicating slower reactions times for gamblers overall (not specific to gambling stimuli). Based on methodological limitations, this study received a quality rating of 63.6% (see Table 1).

Atkins and Sharpe (2003) compared problem gamblers (n = 8) with high (n = 8) and low frequency (n = 8) non-problem gamblers with a modified Stroop task including positive and negative gambling-related, emotional and neutral word stimuli, in addition to a general Stroop task. In contrast to expectation, the sample of problem gamblers within this study demonstrated faster reaction times across conditions, including significantly quicker responses to positive gambling words in comparison to controls (*d* = − 0.735) (reverse interference effect). The authors suggested that the lack of specificity in gambling stimuli may have prevented elicitation of the expected attentional bias effect.

Boyer and Dickerson (2003) sought to replicate and extend the methodology of McCusker and Gettings (1997) using gambling (poker), neutral, and drug-related words, with a focus on exploring impaired control over gambling behaviour rather than clinical diagnosis. They recruited 60 poker machine players, categorised into high control (n = 30) and low control groups (n = 30) based on the Scale of Gambling Choices (SGC) (Baron et al., 1995). They uncovered significantly slower colour naming times for gambling-related words in the low control group as compared to the high control group (*d* = 0.189) with a significant interaction effect between group and condition (*d* = 0.517).

Molde et al. (2010) recruited problem slot-machine gamblers (n = 33) to complete a Stroop task using win-related and neutral pictorial stimuli with both subliminal and supraliminal presentations of gambling stimuli to investigate the unconscious automatic nature of attention. Increased cognitive interference for win-related stimuli was indicated for problem gamblers, who had significantly longer reaction times and reduced accuracy compared to neutral stimuli, and when compared to control subjects (n = 22) (*d* = 0.668).

Lastly, Cutter (2016) designed a gambling-related Stroop task encompassing words related to a broad range of gambling activities alongside negative and neutral words. Participants were categorised according to PGSI scores into problem gamblers (n = 10), moderate problem gamblers (n = 26), low problem gamblers (n = 18), and non-problem gamblers (n = 6). Analysis revealed slower reaction times for gambling words than for neutral words across the whole sample, with no significant interaction between group and condition. Cutter (2016) speculated that this lack of effect may be due to the generic nature of gambling stimuli used within the task, suggesting that specific gambling stimuli related to individual preference may be required.

Overall, studies utilising the addiction Stroop paradigm produced mixed findings. Three reported attentional bias among problem gamblers for gambling-related stimuli (Boyer & Dickerson, 2003; McCusker & Gettings, 1997; Molde et al., 2010), although there was no interaction effects in the research conducted by McCusker and Gettings (1997). One study reported a reverse interference effect (Atkins & Sharpe, 2003), and one study did not reveal any attentional bias effects (Cutter, 2016). Studies ranged in quality assessment ratings from 63.6% (McCusker & Gettings, 1997) to 90.9% (Boyer & Dickerson, 2003; Molde et al., 2010) (Table 1), with the studies with the larger sample sizes (and highest quality ratings) reporting interaction effects (Boyer & Dickerson, 2003; Molde et al, 2010).

### Attentional Blink Task

The ‘attentional blink’ coined by Raymond et al. (1992), refers to the temporary suppression of visual attention mechanisms following allocation of visual attention to ‘important’ stimuli. Attentional blink tasks involve the presentation of two masked stimuli within a rapid serial visual presentation (RSVP) stream, and participants are tasked with identifying the second stimuli. The attentional blink typically results in poor identification of the second stimuli, although this effect is attenuated (blink survival) when this stimulus is personally salient.

Brevers et al. (2011b) utilised the attentional blink paradigm to examine attentional bias in problem gamblers when presented with gambling related and neutral word targets. They found a diminished attentional blink effect (*d* = 0.532) at 200 ms (orienting of attention) for gambling-related words compared to neutral targets in problem gamblers (n = 40), which was not observed in controls (n = 35). A key limitation of the study was the distinct populations from which the experimental and control groups were sampled (casinos vs hospital employees) raising the possibility of confounding factors.

Hudson et al. (2016) sought to expand on the research of Brevers et al. (2011b) by employing additional comparison stimuli alongside neutral items (negative and positive items) and using pictorial rather than word stimuli. They presented targets at either 200 ms or 800 ms to examine attentional bias at orientation and disengagement respectively. They distinguished between high (n = 31) and low risk gamblers (n = 26) in a sample of regular gamblers. In line with PGSI scoring guidelines, participants scoring 0 to 2 were deemed ‘low risk’, however all participants scoring ≥ 3 were included in the ‘high risk’ group. Although the authors reported attentional bias in high-risk gamblers at the level of maintenance/ sustained attention (800 ms) the effect did not quite reach statistical significance (*p* = 0.06). While Hudson et al. (2016) briefly comment on their decision to relax alpha in their results, the lack of clarity in reporting is reflected in the quality assessment rating of this study (72.7%; see Table 1).

### Dual Task Paradigms

Dual task experiments draw upon Cognitive Load Theory (Sweller et al., 1998), which describes the limited capacity of working memory, and the prioritisation of resources when multiple processing demands are imposed. Dual task paradigms therefore involve two tasks occurring concurrently to allow for measurement of performance and allocation of attention under increased cognitive load.

Diskin and Hodgins (1999) employed a dual task paradigm to examine attentional bias in problem gamblers (n = 12) compared to non-problem occasional gamblers (n = 11). Participants were tasked with responding to the presence of an illuminated LED light while playing a video lottery terminal (VLT) game. Although not specifically stated by the authors, the paradigm employed appears to reflect delayed disengagement/ maintenance of attention. Problem gamblers were slower than non-problem gamblers in reacting to light stimuli while playing the VLT game, suggesting a greater narrowing of attention (*d* = 1.179). A key weakness of this study was the absence of baseline performance measurements, leading the authors to replicate the study with a baseline reaction time measurement where responses to LED lights were recorded independently (Diskin & Hodgins, 2001). Problem gamblers (n = 20) and controls (n = 10) did not demonstrate the same overall narrowing of attention in this later study (*d* = 0.052), however a significant interaction between group and condition order was identified (*d* = 1.248). For problem gamblers only, experiencing the baseline condition first resulted in significantly faster response times, which may suggest that the absence of attentional bias in the baseline-first condition may be the result of a practice effect. Additionally, given the intrinsic differences between the baseline and experimental condition in terms of stimulus and difficulty level, the risk of confounding variables cannot be overlooked. While the second study received a greater quality assessment rating (72.7%) than the original study (63.6%), the methodological limitations across both studies are reflected in an average (M) rating of 68.15% (Table 1).

### Lexical Salience Task

Zack and Poulos (2004) developed the Lexical salience task as an amalgamation of the traditional semantic priming task and pharmacological priming in order to investigate the priming effect of a psychostimulant (oral D-amphetamine, AMPH) on the motivation to gamble in problem gamblers (n = 10), who were compared against comorbid gambler-drinkers (n = 6), problem drinkers (n = 8), and healthy controls (n = 12). They employed a modified rapid reading task encompassing five semantic domains (Gambling, Alcohol, Positive Affect, Negative Affect, Neutral). The task required participants to read aloud a series of randomised target (gambling) and control words under AMPH and placebo conditions, with faster reading times denoting greater attention due to motivational salience. In the placebo condition (without psychostimulant), problem gamblers did not demonstrate a significant difference in reading speed across word categories.

The authors conducted a further study examining the priming effect of dopamine D2 agonist haloperidol on performance on a lexical salience task (Zack & Poulos, 2007), comparing reading reaction times of problem gamblers (n = 20) with controls (n = 18) on gambling and neutral words. Consistent with their earlier study, the authors did not discover any significant differences in reading reaction times in the placebo condition. It is of note that both of these studies employed small samples which were not justified in terms of statistical power, although overall quality assessment ratings were good (81.8%; see Table 1).

### Flicker-induced Change Blindness Paradigm

As defined by Attwood et al. (2018), ‘change blindness is a phenomenon of visual perception that occurs when a stimulus undergoes a change without this being noticed by its observer.’ (p.151). This phenomenon has been discovered in various contexts, including eyewitness identification (Fitzgerald et al., 2016), insomnia (Marchetti et al., 2006), and alcohol intoxication (Colflesh & Wiley, 2013).

Brevers et al. (2011a) utilised a flicker-induced change blindness paradigm, in which ‘two images differing in only one aspect were repeatedly flashed on the screen until the participant was able to report the changing item’ (neutral/gambling-related). Measures of change detection latency revealed significant attentional biases toward gambling-related visual cues (e.g. poker chips) in problem gamblers (n = 22) compared to controls (n = 35) (*d* = 0.76). Additional eye-gaze tracking data revealed that problem gamblers directed initial eye movements towards gambling stimuli more than neutral stimuli (*d* = 1.09), demonstrated more gaze fixations on gambling stimuli (*d* = 0.577), and looked at them for longer (*d* = 0.734). Taken together, Brevers et al. (2011a) concluded that the behavioural and eye-tracking data indicated attentional bias at both orientation and maintenance stages of attention in problem gamblers. This study received a quality assessment rating of 81.8%, although was limited by the lack of an *a prior*i power analysis and the absence of inclusion of gambling frequency as a potential confounding variable (see Table 1).

### EEG Cue-Reactivity

Event related potentials (ERP’s) represent a direct measure of attentional bias through measurement of neural activity in response to stimuli. Higher amplitude ERP components during stimulus processing denote attentional bias, with early ERP components thought to indicate bias at orientation, and late positive waves understood to signify delayed disengagement (Field et al., 2009).

Wölfling et al. (2011) examined emotional processing of gambling and non-gambling stimulus material (positive, negative and neutral) in problem gamblers (n = 15) and non-gambling controls (n = 15) using an EEG cue-reactivity paradigm. Late positive potentials (LPP’s) were measured, based on the premise that larger LPP’s are elicited in response to high arousal stimuli which hold greater emotional significance. Non-gambling stimuli were processed similarly across the two groups, however problem gamblers showed significantly larger LPP’s in response to gambling stimuli than controls (*d* = 1.373) indicating attentional bias in the maintenance of attention. This study received a quality assessment rating of 81.8% (see Table 1).

### Approach Avoidance Task

Boffo et al. (2018) adapted the approach avoidance task developed by Rinck and Becker (2007) in their research into fear of spiders. The task requires participants to either approach (“pull”) or avoid (“push”) neutral and target stimuli using a joystick or keyboard keys, appearing to reflect attentional bias at orientation of attention. Boffo et al. (2018) adapted this task to examine attentional bias in problem gamblers using gambling-related and neutral pictorial stimuli in a sample of moderate to high-risk gamblers (n = 22) and non-problem gamblers (n = 26). Approach bias scores were calculated by subtracting median reaction times in each stimulus category for both approach and avoid trials, where a faster ‘pull’ response to gambling stimuli relative to neutral stimuli indicates a stronger approach tendency. Analysis revealed a greater approach bias towards gambling stimuli in moderate to high-risk gamblers relative to non-problem gamblers (*d* = 0.38). This study received a quality assessment rating of 90.9% (Table 1).

### Posner Paradigm

The Posner paradigm (Posner, 1980) requires participants to indicate the location of a target stimulus in one of two locations following a visual cue, which either appears in the same location as the visual stimulus (valid trial), or in the other location (invalid trial). Customarily, response times on the Posner task are quicker for valid trials, in line with the hypothesis that cues orient visual attention. In addiction research, attentional bias for substance-related cues is established by shorter reaction times to probes that appear in the location of substance-related stimuli as opposed to probes which replace neutral/control stimuli (Field et al., 2009). Ciccarelli and colleagues (2016a, 2016b, 2019, 2020) modified the Posner task for use with a gambling population, examining attentional bias at both orientation and maintenance of attention by manipulating the length of stimulus presentation. It is of note that none of the studies within this paradigm provided an *a prior*i power analysis, nor did they match for gambling frequency as a potential confounding variable. All four studies subsequently received quality assessment ratings of 81.8% (see Table 1).

Ciccarelli et al. (2016a) employed a modified Posner task to investigate attentional bias in problem gamblers (n = 25), non-problem gamblers (n = 25) and abstinent ‘pathological gamblers’ who had a DSM-V diagnosis of Gambling Disorder and were undergoing treatment (n = 25). They used gambling and neutral images as ‘cues’ for the target stimulus and calculated facilitation and disengagement biases. Problem gamblers demonstrated a facilitation bias at 100 ms (*d* = 1.028) but no disengagement bias, and abstinent problem gamblers were slower to detect neutral stimuli following presentation of gambling cues in valid trials only (attentional avoidance).

Ciccarelli et al. (2016b) repeated this task with a sample of 108 problem and non-problem gamblers with consistent results. They found that problem gamblers (n = 54) were faster to respond to gambling-related stimuli when presented at 100 ms (initial orientation) (*d* = 0.865), whereas non-problem gamblers (n = 54) did not differ in their response times between neutral and gambling-related stimuli. The same authors conducted a further study (Ciccarelli et al., 2020) in which the modified Posner task was completed by 28 problem gamblers and 42 non-problem gamblers. In accordance with their earlier studies, Ciccarelli et al. (2020) reported facilitation bias for gambling-related stimuli at 100 ms in problem gamblers (*d* = 0.701) with no bias at disengagement (500 ms).

Ciccarelli et al. (2019) replicated this task with adolescent problem gamblers (age 16–20; *M* = 17.54 years; *SD* = 0.89), producing interesting results. In contrast to adult problem gamblers, adolescents demonstrated facilitation bias at 500 ms, demonstrating bias at the maintenance of attention rather than initial orientation (*d* = 0.742). The authors postulated that the findings support a conscious and intentional orientation of attention to gambling stimuli in adolescents, as compared to an unconscious automatic process in adults as familiarity with gambling stimuli is greater.

### Eye-Gaze Tracking

Eye-gaze tracking involves the use of a computer or other video device to record eye movements as a direct measure of attention. It allows continuous measurement of eye movements in response to stimuli, both spatially and temporally to identify fixations and saccades (Skinner et al., 2018). The average (M) quality assessment rating across the three studies conducted within this paradigm was 84.8% (see Table 1).

McGrath et al. (2021) utilised eye-gaze tracking to measure attentional bias in undergraduate students categorised by PGSI scores into no risk (n = 38), low risk (n = 24), and moderate/high risk groups (n = 25). Participants were presented with 25 pairs of images (neutral/gambling) along with 31 pairs of neutral images (filler trials). Analysis revealed no difference in initial orientation to stimuli (gambling vs neutral), however the moderate/high risk group demonstrated sustained attentional bias during the last 4 s of the 8 s image presentations compared to the no risk (*d* = 1.361) and low risk (*d* = 0.638) groups.

Kim et al. (2021) employed a similar methodology in their examination of attentional bias in Electronic Gaming Machine (EGM) gamblers. Participants were presented with four images per trial, which consisted of either three neutral images and one EGM image (experimental trials), or four neutral images (filler trials). Participants were classified as either non-gambling disorder (non-GD, n = 52) or gambling disorder (GD, n = 25) EGM players based on PGSI scores (GD =  ≥ 5), alongside a control group of non-gamblers (n = 60). Both non-GD and GD EGM players demonstrated attentional bias towards EGM images (orientation of attention), with a significantly larger effect present in GD players compared to both non-GD players (*d* = 1.38) and controls (*d* = 2.55). A further study by Kim et al. (2022) using the same experimental task found that PGSI scores were a significant predictor of attentional bias (*d* = 1.023).

### Visual Probe Task

The visual probe task has been employed in research into substance use for more than two decades. The task involves the simultaneous presentation of a substance-related and neutral visual stimulus, followed by a visual probe which appears in the location of one of the previous stimuli. Participants are required to respond as quickly as possible to the appearance of the probe, and reaction times form the basis for analysis, where faster responses to probes appearing in the location of the substance-related stimuli indicates attentional bias (Field & Cox, 2008).

Vizcaino et al. (2012) used gambling and neutral images in a visual probe task with ‘pathological gamblers’ (n = 23) recruited from an outpatient gambling treatment clinic. In this study, pathological gamblers demonstrated attentional bias at the maintenance of attention for gambling-related stimuli (*d* = 0.815) which was not observed in controls (n = 21), however there was not a significant correlation between attentional bias and gambling severity as measured by SOGS scores. The authors attributed the absence of a correlation to the lack of variation in SOGS scored among pathological gamblers and highlighted the binary nature of the sample as a key weakness of the research. As non-problem gamblers were not represented in the sample, the presence of attentional bias in pathological gamblers was not established as distinct from potential bias in non-problem social gamblers. This study received a quality assessment rating of 81.8%.

## Discussion

Significant attentional bias effects for gambling-related stimuli in problem gamblers was demonstrated in 16 of the 22 studies examined. Five of the 22 studies utilised direct measures (ERP, eye-gaze tracking) (Brevers et al., 2011a, 2011b; Kim et al., 2021, 2022; McGrath et al., 2021; Wölfling et al., 2011), all of which reported significant attentional bias in problem gamblers. Given that almost all of the studies reviewed operationalised gambling severity using the PGSI or SOGS, differences between paradigms cannot be accounted for as a function of different measures of gambling severity.

Differences in attentional bias effects across studies can be observed at a paradigm level. Zack and Poulos (2004, 2007) found no attentional bias using a lexical salience task, however there is still a lack of clarity regarding the involvement of attentional processes in this experimental paradigm. The authors refer to Robinson and Berridge's (1993) theory of incentive salience, which suggests that faster reading times may reflect increased salience or motivational relevance, but the specific relationship with attentional bias remains unclear. Consequently, there are doubts regarding the effectiveness of this method as a measure of attentional bias. Studies using the Stroop Task produced mixed findings, with three of five studies noting an attentional bias effect in problem gamblers for gambling-related stimuli. Where an effect was found in the expected direction, studies utilised specific gambling stimuli related to activity preference (Boyer & Dickerson, 2003; McCusker & Gettings, 1997; Molde et al., 2010), whereas those employing non-specific gambling stimuli found either no attentional bias effect (Cutter, 2016), or the effect was observed in the opposite direction (Atkins & Sharpe, 2003).

Diskin and Hodgins (1999) reported attentional bias using a dual task paradigm, however the absence of a baseline performance measure or control condition call into question the validity of the results. The same effect was not found in their later study (2001) following introduction of a baseline condition. While Brevers et al. (2011b) demonstrated a significant attentional bias effect for gambling-related words in problem gamblers using an attentional blink paradigm, the same results were not demonstrated by Hudson et al. (2016) with effects falling short of statistical significance.

The remaining experimental paradigms consistently revealed attentional bias among problem gamblers for gambling related stimuli, with Ciccarelli et al., (2016a, 2016b, 2019, 2020) reporting large effect sizes on four studies employing a modified Posner task.

The reviewed studies provide evidence for attentional bias at both orientation and maintenance of attention, with eight studies producing effects relevant to attention orientation (Boffo et al., 2018; Boyer & Dickerson, 2003; Brevers et al., 2011b; Ciccarelli et al., 2016a, 2016b, 2020; McCusker & Gettings, 1997; Molde et al., 2010) and seven reporting attentional bias at the maintenance level (Diskin & Hodgins, 1999; Wölfling et al., 2011; Hudson, 2016; Ciccarelli et al., 2019; McGrath et al., 2021; Vizcaino et al., 2012; Kim et al., 2021, 2022). The study by Brevers et al. (2011a) concluded that effects indicated attentional bias at both orientation and maintenance of attention.

The majority of studies did not use experimental methods which assess for both orientation and maintenance and as such it is not possible to determine whether an attentional bias effect would have been observed at both stages. The five studies reporting significant effects through implementation of such methods yielded varying results. Ciccarelli et al., (2016a, 2016b, 2020) consistently found attentional bias at the stage of attentional orientation in adult problem gamblers, however reported bias at the level of maintenance of attention in adolescent gamblers (Ciccarelli et al., 2019). The authors suggest that this may reflect a move from conscious intentional attentional orientation in the initial stages of problem gambling, to a more automatic unconscious attentional bias in line with increased familiarity with gambling. In contrast, Brevers et al. (2011a) assessed for both orientation and maintenance of attention using a combination of direct and indirect measures (eye gaze tracking and change detection latency) and observed attentional bias at both orientation and maintenance.

Two main quality limitations were identified across studies. Only three of the 22 studies justified their sample size through a priori power analysis (Kim et al., 2021, 2022; Molde et al., 2010) Therefore, in studies where attentional bias was not found, this may be reflective of low statistical power rather than the absence of an effect (Abraham & Russell, 2008). Secondly, only two studies took into account gambling frequency as a possible confounding variable (Boffo et al., 2018; Boyer & Dickerson, 2003). Therefore, it is plausible that any differences observed between groups may be attributed to or moderated by gambling frequency where this was not controlled for. Overall, studies ranged in quality ratings from 63.6% to 90.9%, with the average (M) quality rating across studies at 80.6%.

### How do findings align with other studies on substance abuse/attentional bias in non-gambling contexts?

Attentional bias has been widely observed in both anxiety and depression (Lichtenstein-Vidne et al., 2017), where an increased allocation of attention to threat-based or other negative stimuli is widely regarded as central in both the development and maintenance of symptoms. This association has also been found to extend to other psychological disorders such as eating disorders (e.g. Shafran et al, 2007), and has a compelling evidence base in addiction and substance use research (e.g. Field & Cox, 2008; MacLean et al, 2018; O’Neill et al., 2020). In summary, this review indicates that the findings in the problem gambling field are generally consistent with those in the substance abuse field.

### Limitations

By nature of adherence to a stringent systematic search protocol, this systematic review is limited to studies meeting specific eligibility criteria and therefore does not consider all studies relating to attention in gambling. For example, two studies were excluded from the current review due to lack of differentiation between problem and non-problem gamblers in the analysis.

Additionally, it was not possible to conduct a meta-analysis as part of the review due to methodological heterogeneity across studies, and as such effect sizes are only available on an individual basis and it is not possible to provide an overall statistical synthesis of reported effects.

Similarly, conclusions drawn are limited by the lack of available studies and heterogeneity across paradigms. Significant variability in experimental methods presents a challenge in making comparisons, and as such the outcomes of current review are more heavily focussed on recommendations for future research rather than drawing meaningful conclusions.

### Implications for Treatment of Problem Gambling

Attentional bias modification (ABM) has been used in the treatment of anxiety disorders, aiming to reduce pathology by diminishing attentional bias to threat (Mogg et al., 2017). Given the potential role of attentional bias as a maintaining factor in addiction and substance use disorders, the utility of ABM interventions has also been explored as a tool for reducing alcohol consumption (Fadardi & Cox, 2009) and targeting opiate addiction (e.g. Charles et al., 2015). Heitmann et al. (2018) conducted a systematic review of ABM interventions in substance use disorders, reporting inconsistent results across studies in relation to changes in substance-related symptoms. Based on the available evidence, the authors concluded that multi-session ABM interventions may be clinically useful in targeting symptom reduction in addictive behaviour, however emphasised the need for further research.

Given the significant parallels between substance misuse and problem gambling, there has been an emerging interest in exploring the feasibility and effectiveness of ABM interventions in problem gambling. Research into this area is in its infancy with regards to the evidence base, with only one published ABM pilot trial (Wittekind et al., 2019), and one study protocol (Boffo et al., 2017). The pilot trial conducted by Wittekind et al. (Wittekind et al., 2019) explored the efficacy of an Approach Bias Modification (AppBM) intervention in reducing gambling-related symptoms in problem slot-machine gamblers. The AppBM was a training task based on the approach-avoidance task (Boffo et al., 2018), where gambling (slot-machine) related pictures had to be pushed and all neutral pictures had to be pulled. Participants were randomly assigned to the AppBM or the Sham condition, in which push and pulls were 50:50 for both stimulus categories. Both groups showed a similar reduction in gambling-related symptoms, which the authors postulated may be due to expectancy effects.

Given the significantly limited evidence base for ABM interventions at present, it is not possible to draw conclusions in relation to their potential clinical impact in the treatment of problem gambling. However, the results of the current review provide robust support for the presence of attentional bias in problem gambling maintenance, and as such it is likely to be beneficial to further explore interventions of this type.

## Conclusions and Recommendations

In line with the defined objectives, this review has outlined attentional bias effects across included studies, provided a quality assessment, and considered the evidence for attentional bias at both orientation and maintenance of attention. Note that the current review encompasses several paradigms that were absent from the 2013 review conducted by Honsi et al. For instance, the Posner, Visual Probe, and Approach-Avoidance paradigms were previously unexplored in gambling research but have now yielded compelling evidence supporting the existence of attentional bias in problem gamblers. Notably, the Posner paradigm, as employed by Ciccarelli and colleagues, provided data supporting potential distinctions in attentional bias between adolescents and adults. Furthermore, eye-gaze tracking, which serves as a direct measure of attentional bias, had not been employed beyond the Brevers et al. (2011a) study, with the additional three studies included in the present review (McGrath et al., 2021; Kim et al., 2021, 2022) demonstrating consistent attentional bias effects among problem gamblers. Overall the findings of this review support the role of attentional bias as a potential maintaining factor in problem gambling behaviour, in line with evidence for substance addiction. While a small proportion of studies did not report an attentional bias effect, this may plausibly be associated with methodological shortcomings or insufficient statistical power. As such, it is recommended that future studies prioritise power analyses to ensure sufficient recruitment of participants.

Methodologically, we advocate for the use of gambling specific stimuli related to activity preference in line with the observed findings in gambling Stroop tasks. Additionally, future studies should endeavour to control for gambling frequency as a potential confounding variable, and further investigation into the role of gambling frequency in attentional bias is necessitated.

Despite increasingly robust support for the role of attentional bias in problem gambling there is still a limited evidence base for the phenomena, particularly at a paradigm level. As such, we advocate for replication of studies with the inclusion of various control groups including abstinent problem gamblers to allow examination of variations in attentional bias across the gambling spectrum. We also recommend further investigation of attentional bias utilising direct measures, which are widely regarded as more sensitive than indirect behavioural measures (Field et al., 2014) and are less vulnerable to confounding variables such as motor speed in measures of reaction time (Sippel et al., 2022).

There remains a lack of clarity around the specific nature of attentional allocation (orientation/ maintenance), necessitating further examination through manipulation of stimulus presentation times. Optimally, studies will incorporate stimulus presentations at different time points to allow simultaneous examination of orientation and facilitation, and permit identification of bias at both time points where this exists. Such an approach has the potential to provide valuable insights into the cognitive mechanisms that drive attentional bias and to further elucidate the complex interplay between attentional processes and gambling behaviour.

Furthermore, in light of the divergent findings concerning problem gambling behaviour in adolescents versus adults, as presented in the seminal works of Ciccarelli and colleagues (2016a, 2016b, 2019, 2020), it is imperative to conduct further research to delve into the intricate dynamics of attentional bias and the temporal aspects of gambling engagement.

In summary, the review supports attentional bias as a potential factor in the maintenance of problem gambling behaviour. Future studies should prioritize power analyses, gambling-specific stimuli, replication with control groups, and direct measures to examine attentional bias. Additionally, investigations should focus on the specific nature of attention allocation and its relationship with duration of gambling career. Overall, further research is necessary to understand the interplay between attentional processes and gambling behaviour.

## Data Availability

Data sharing not applicable to this article as no datasets were generated or analysed during the current study.

## Introduction

1. Were the aims/objectives of the study clear? (AXIS, 2016).

## Methods

2. Was the sample size justified? (AXIS, 2016).
3. Was membership in a ‘problem gambling’ group established through use of a reputable screening tool (e.g. PGSI/SOGS/DSM-V)? (Adapted from SIGN (2012): ‘*Cases are clearly defined and differentiated from controls*’).
4. Were the gambling and control group(s) matched for gambling frequency as a confounding variable? (Adapted from CASP (2018): ‘*Have the authors identified all important confounding factors?’*).

*Rationale: Gambling frequency was specified as a confounding variable due to the positive association with problem gambling* (e.g. Mazar et al., 2020)* and evidence for the predictive relationship of frequency in attentional bias to gambling cues (*Grant & Bowling, 2015*). Where gambling frequency is not controlled for, it is not possible to distinguish between differences due to frequency or problems, or both.*

5. Were additional conditions included to offer a comparison to performance in gambling conditions?

*Rationale: This question was created to assess internal validity – the absence of control conditions as a basis for comparison would make it impossible to draw conclusions about the impact of group membership (e.g. problem gamblers vs controls) (*Torday & Baluška, 2019*).*

6. Were the experimental and control groups sampled from the same population? (Adapted from CASP (2020): ‘*Were the study groups similar at the start of the randomised controlled trial?’’.*
7. Was the selection process likely to select subjects/participants that were representative of the target/reference population under investigation? (AXIS, 2016).
8. Were the outcome variables measured appropriate to the aims of the study? (AXIS, 2016).
9. Is it clear what was used to determined statistical significance and/or precision estimates? (e.g. *p*-values, confidence intervals) (AXIS, 2016).

## Results

10 Were the basic data adequately described? (AXIS, 2016).
11 Were the results presented for all the analyses described in the methods? (AXIS, 2016).

## References

[CR1] Abraham, W., & Russell, D. (2008). Statistical power analysis in psychological research. *Social and Personality Psychology Compass,**2*, 283–301. 10.1111/j.1751-9004.2007.00052.x 10.1111/j.1751-9004.2007.00052.x

[CR2] American Psychiatric Association. (2013). *Diagnostic and statistical manual of mental disorders: Diagnostic and statistical manual of mental disorders* (5th ed.). American Psychiatric Association.

[CR3] Atkins, G., & Sharpe, L. (2003). Cognitive biases in problem gambling. *Gambling Research: Journal of the National Association for Gambling Studies,**15*(2), 35.

[CR4] Attwood, J. E., Kennard, C., Harris, J., Humphreys, G., & Antoniades, C. A. (2018). A comparison of change blindness in real-world and on-screen viewing of museum artefacts. *Frontiers in Psychology,**9*, 151. 10.3389/fpsyg.2018.00151 29503625 10.3389/fpsyg.2018.00151 PMC5820331

[CR5] Baron, E., Dickerson, M., & Blaszcynski, A. (1995). The scale of gambling choices: Preliminary development of an instrument to measure impaired control of gambling behaviour. In J. O’Connor (Ed.), *High stakes in the nineties* (pp. 153–167). Curtin University Press.

[CR6] Boffo, M., Willemen, R., Pronk, T., Wiers, R. W., & Dom, G. (2017). Effectiveness of two web-based cognitive bias modification interventions targeting approach and attentional bias in gambling problems: Study protocol for a pilot randomised controlled trial. *Trials,**18*(1), 452. 10.1186/s13063-017-2190-2 28974265 10.1186/s13063-017-2190-2 PMC5627491

[CR7] Boffo, M., Smits, R., Salmon, J. P., Cowie, M. E., de Jong, D. T. H. A., Salemink, E., Collins, P., Stewart, S. H., & Wiers, R. W. (2018). Luck, come here! Automatic approach tendencies toward gambling cues in moderate- to high-risk gamblers. *Addiction,**113*, 289–298. 10.1111/add.14071 29055971 10.1111/add.14071

[CR8] Boyer, M., & Dickerson, M. (2003). Attentional bias and addictive behaviour: Automaticity in a gambling- specific modified stroop task. *Addiction,**98*(1), 61–70. 12492756 10.1046/j.1360-0443.2003.00219.x

[CR9] Brevers, D., Cleeremans, A., Bechara, A., Laloyaux, C., Kornreich, C., Verbanck, P., & Noël, X. (2011a). Time course of attentional bias for gambling information in problem gambling. *Psychology of Addictive Behaviors,**25*(4), 675–682. 10.1037/a0024201 21688874 10.1037/a0024201 PMC3792789

[CR10] Brevers, D., Cleeremans, A., Tibboel, H., Bechara, A., Kornreich, C., Verbanck, P., & Noël, X. (2011b). Reduced attentional blink for gambling-related stimuli in problem gamblers. *Journal of Behavior Therapy and Experimental Psychiatry,**42*(3), 265–269. 10.1016/j.jbtep.2011.01.005 21349247 10.1016/j.jbtep.2011.01.005

[CR11] Charles, M., Wellington, C. E., Mokrysz, C., Freeman, T. P., O’Ryan, D., & Curran, H. V. (2015). Attentional bias and treatment adherence in substitute-prescribed opiate users. *Addictive Behaviors,**46*, 100–105. 10.1016/j.addbeh.2015.03.017 25838001 10.1016/j.addbeh.2015.03.017

[CR12] Ciccarelli, M., Nigro, G., Griffiths, M. D., Cosenza, M., & D’Olimpio, F. (2016a). Attentional bias in non-problem gamblers, problem gamblers, and abstinent pathological gamblers: An experimental study. *Journal of Affective Disorders,**206*, 9–16. 27455353 10.1016/j.jad.2016.07.017

[CR13] Ciccarelli, M., Nigro, G., Griffiths, M. D., Cosenza, M., & D’Olimpio, F. (2016b). Attentional biases in problem and non-problem gamblers. *Journal of Affective Disorders,**198*, 135–141. 10.1016/j.jad.2016.03.009 27016656 10.1016/j.jad.2016.03.009

[CR14] Ciccarelli, M., Cosenza, M., Griffiths, M. D., Nigro, G., & D’Olimpio, F. (2019). Facilitated attention for gambling cues in adolescent problem gamblers: An experimental study. *Journal of Affective Disorders,**252*, 39–46. 10.1016/j.jad.2019.04.012 30978623 10.1016/j.jad.2019.04.012

[CR15] Ciccarelli, M., Griffiths, M. D., Cosenza, M., Nigro, G., & D’Olimpio, F. (2020). Disordered gambling and attentional bias: The mediating role of risk-taking. *Journal of Affective Disorders,**272*, 496–500. 32553393 10.1016/j.jad.2020.03.144

[CR16] Colflesh, G. J. H., & Wiley, J. (2013). Drunk, but not blind: The effects of alcohol intoxication on change blindness. *Consciousness and Cognition: An International Journal,**22*, 231–236. 10.1016/j.concog.2013.01.001 10.1016/j.concog.2013.01.001 23357240

[CR17] Cox, W. M., Klinger, E., & Fadardi, J. S. (2016). Nonconscious motivational influences on cognitive processes in addictive behaviors. In N. Heather & G. Segal (Eds.), *Addiction and choice: Rethinking the relationship. *Oxford University Press. 10.1093/acprof:oso/9780198727224.003.0015

[CR18] Critical Appraisal Skills Programme. (2018). *CASP Cohort Study Checklist.* https://casp-uk.net/casp-tools-checklists/

[CR19] Critical Appraisal Skills Programme. (2020). *CASP Cohort Study Randomised Controlled Trial Standard Checklist.* https://casp-uk.net/casp-tools-checklists/

[CR20] Cutter, R. (2016). A longitudinal study mapping changes in explicit and implicit measures of gambling behaviour. Retrieved November 7, 2022, from https://www.begambleaware.org/sites/default/files/2020-12/Richard%20Cutter%20PhD%20010317.pdf

[CR21] Diskin, K. M., & Hodgins, D. C. (1999). Narrowing of attention and dissociation in pathological video lottery gamblers. *Journal of Gambling Studies,**15*(1), 17–28. 12766452 10.1023/A:1023062912062

[CR22] Diskin, K. M., & Hodgins, D. C. (2001). Narrowed focus and dissociative experiences in a community sample of experienced video lottery gamblers. *Canadian Journal of Behavioural Science,**33*(1), 58–64. 10.1037/h0087128

[CR23] Downes, M. J., Brennan, M. L., Williams, H. C., & Dean, R. S. (2016). Development of a critical appraisal tool to assess the quality of cross-sectional studies (AXIS). *British Medical Journal Open,**6*(12), e011458. 10.1136/bmjopen-2016-011458 10.1136/bmjopen-2016-011458 PMC5168618 27932337

[CR24] Fadardi, J. S., & Cox, W. M. (2009). Reversing the sequence: Reducing alcohol consumption by overcoming alcohol attentional bias. *Drug and Alcohol Dependence,**101*(3), 137–145. 10.1016/j.drugalcdep.2008.11.015 19193499 10.1016/j.drugalcdep.2008.11.015

[CR26] Fernández-Calderón, F., Lozano, O. M., Moraleda-Barreno, E., Lorca-Marín, J. A., & Díaz-Batanero, C. (2021). Initial orientation vs maintenance of attention: Relationship with the severity of dependence and therapeutic outcome in a sample of cocaine use disorder patients. *Addictive Behaviours,**116*, 106834. 10.1016/j.addbeh.2021.106834 10.1016/j.addbeh.2021.106834 33503505

[CR27] Ferris, J., & Wynne, H. J. (2001). *The Canadian problem gambling index final report*. Canadian Centre on Substance Abuse.

[CR28] Field, M., & Cox, W. M. (2008). Attentional bias in addictive behaviors: A review of its development, causes, and consequences. *Drug and Alcohol Dependence,**97*(1–2), 1–20. 18479844 10.1016/j.drugalcdep.2008.03.030

[CR29] Field, M., Munafò, M. R., & Franken, I. H. (2009). A meta-analytic investigation of the relationship between attentional bias and subjective craving in substance abuse. *Psychological Bulletin,**135*(4), 589–607. 10.1037/a0015843 19586163 10.1037/a0015843 PMC2999821

[CR30] Field, M., Marhe, R., & Franken, I. (2014). The clinical relevance of attentional bias in substance use disorders. *CNS Spectrums,**19*(3), 225–230. 10.1017/S1092852913000321 23663386 10.1017/S1092852913000321

[CR31] Fitzgerald, R., Oriet, C., & Price, H. (2016). Change blindness and eyewitness identification: Effects on accuracy and confidence. *Legal and Criminological Psychology,**21*, 189–201. 10.1111/lcrp.12044 10.1111/lcrp.12044

[CR32] Grant, L. D., & Bowling, A. C. (2015). Gambling attitudes and beliefs predict attentional bias in non-problem gamblers. *Journal of Gambling Studies,**31*(4), 1487–1503. 24871298 10.1007/s10899-014-9468-z

[CR33] Heitmann, J., Bennik, E. C., van Hemel-Ruiter, M. E., & de Jong, P. J. (2018). The effectiveness of attentional bias modification for substance use disorder symptoms in adults: A systematic review. *Systematic Reviews,**7*(1), 160. 10.1186/s13643-018-0822-6 30316302 10.1186/s13643-018-0822-6 PMC6186103

[CR34] Hønsi, A., Mentzoni, R., Molde, H., & Pallesen, S. (2013). Attentional bias in problem gambling: A systematic review. *Journal of Gambling Studies,**29*(3), 359–375. 10.1007/s10899-012-9315-z 22644097 10.1007/s10899-012-9315-z

[CR35] Hudson, A., Olatunji, B. O., Gough, K., Yi, S., & Stewart, S. H. (2016). Eye on the prize: High-risk gamblers show sustained selective attention to gambling cues. *Journal of Gambling Issues,**34*, 100–119. 10.4309/jgi.2016.34.6 10.4309/jgi.2016.34.6

[CR36] Kim, H. S., Sears, C. R., Hodgins, D. C., Ritchie, E. V., Kowatch, K. R., & McGrath, D. S. (2021). Gambling-related psychological predictors and moderators of attentional bias among electronic gaming machine players. *Psychology of Addictive Behaviors: Journal of the Society of Psychologists in Addictive Behaviors,**35*(8), 961–973. 10.1037/adb0000716 33749291 10.1037/adb0000716

[CR37] Kim, H. S., Ritchie, E. V., Sears, C. R., Hodgins, D. C., Kowatch, K. R., & McGrath, D. S. (2022). Affective impulsivity moderates the relationship between disordered gambling severity and attentional bias in electronic gaming machine (EGM) players. *Journal of Behavioral Addictions,**11*(2), 386–395. 10.1556/2006.2022.00043 35895477 10.1556/2006.2022.00043 PMC9295233

[CR38] Klinger, E., & Cox, W. M. (2004). Motivation and the theory of current concern. In W. M. Cox & E. Klinger (Eds.), *Handbook of motivational counseling: Concepts, approaches, and assessment* (pp. 3–27). Wiley.

[CR39] Leeman, R. F., & Potenza, M. N. (2012). Similarities and differences between pathological gambling and substance use disorders: A focus on impulsivity and compulsivity. *Psychopharmacology (berl),**219*(2), 469–490. 10.1007/s00213-011-2550-7 22057662 10.1007/s00213-011-2550-7 PMC3249521

[CR40] Lesieur, H. R., & Blume, S. B. (1987). The South Oaks Gambling Screen (SOGS): A new instrument for the identification of pathological gamblers. *American Journal of Psychiatry,**144*, 1184–1188. 3631315 10.1176/ajp.144.9.1184

[CR41] Lichtenstein-Vidne, L., Okon-Singer, H., Cohen, N., Todder, D., Aue, T., Nemets, B., & Henik, A. (2017). Attentional bias in clinical depression and anxiety: The impact of emotional and non-emotional distracting information. *Biological Psychology,**122*, 4–12. 10.1016/j.biopsycho.2016.07.012 27422409 10.1016/j.biopsycho.2016.07.012

[CR42] MacLean, R. R., Sofuoglu, M., Brede, E., Robinson, C., & Waters, A. J. (2018). Attentional bias in opioid users: A systematic review and meta-analysis. *Drug and Alcohol Dependence,**191*, 270–278. 10.1016/j.drugalcdep.2018.07.012 30157467 10.1016/j.drugalcdep.2018.07.012

[CR75] Marchetti, L. M., Biello, S. M., Broomfield, N. M., Macmahon, K. M., & Espie, C. A. (2006). Who is pre-occupied with sleep? A comparison of attention bias in people with psychophysiological insomnia, delayed sleep phase syndrome and good sleepers using the induced change blindness paradigm. *Journal of Sleep Research*, *15*(2), 212–221. 10.1111/j.1365-2869.2006.00510 16704577 10.1111/j.1365-2869.2006.00510

[CR43] Marks, K. R., Roberts, W., Stoops, W. W., Pike, E., Fillmore, M. T., & Rush, C. R. (2014). Fixation time is a sensitive measure of cocaine cue attentional bias. *Addiction,**109*, 1501–1508. 10.1111/add.12635 24894879 10.1111/add.12635 PMC4612370

[CR76] Mazar, A., Zorn, M., Becker, N., & Volberg, R. A. (2020). Gambling formats, involvement, and problem gambling: Which types of gambling are more risky? *BMC Public Health*, *20*(1), 711. 10.1186/s12889-020-08822-2 32423451 10.1186/s12889-020-08822-2 PMC7236368

[CR44] McCusker, C. G., & Gettings, B. (1997). Automaticity of cognitive biases in addictive behaviours: Further evidence with gamblers. *British Journal of Clinical Psychology,**36*(4), 543–554. 9403145 10.1111/j.2044-8260.1997.tb01259.x

[CR45] McGrath, D. S., Sears, C. R., Fernandez, A., & Dobson, K. S. (2021). Attentional biases in low-risk and high-risk gamblers and the moderating effect of daily psychosocial stress. *Addiction Research & Theory,**29*(2), 166–174. 10.1080/16066359.2020.1762867

[CR46] McShane, B. B., & Böckenholt, U. (2017). Single-paper meta-analysis: Benefits for study summary, theory testing, and replicability. *Journal of Consumer Research,**43*(6), 1048–1063. 10.1093/jcr/ucw085

[CR47] Mogg, K., Waters, A. M., & Bradley, B. P. (2017). Attention bias modification (ABM): Review of effects of multisession ABM training on anxiety and threat-related attention in high-anxious individuals. *Clinical Psychological Science: A Journal of the Association for Psychological Science,**5*(4), 698–717. 10.1177/2167702617696359 28752017 10.1177/2167702617696359 PMC5513441

[CR48] Moher, D., Liberati, A., Tetzlaff, J., Altman, D. G., PRISMA Group*. (2009). Preferred reporting items for systematic reviews and meta-analyses: the PRISMA statement. *Annals of Internal Medicine,**151*(4), 264–269. 19622511 10.7326/0003-4819-151-4-200908180-00135

[CR49] Molde, H., Pallesen, S., Sætrevik, B., Hammerborg, D., Laberg, J., & Johnsen, B. H. (2010). Attentional biases among pathological gamblers. *International Journal of Gambling Studies*. 10.1080/14459791003652501 10.1080/14459791003652501

[CR50] O’Neill, A., Bachi, B., & Bhattacharyya, S. (2020). Attentional bias towards cannabis cues in cannabis users: A systematic review and meta-analysis. *Drug and Alcohol Dependence,**206*, 107719. 10.1016/j.drugalcdep.2019.107719 31753732 10.1016/j.drugalcdep.2019.107719

[CR51] Pallanti, S., Marras, A., & Makris, N. (2021). a research domain criteria approach to gambling disorder and behavioral addictions: Decision-making, response inhibition, and the role of cannabidiol [hypothesis and theory]. *Frontiers in Psychiatry*. 10.3389/fpsyt.2021.634418 34603091 10.3389/fpsyt.2021.634418 PMC8484302

[CR52] Posner, M. I. (1980). Orienting of attention. *The Quarterly Journal of Experimental Psychology,**32*(1), 3–25. 10.1080/00335558008248231 7367577 10.1080/00335558008248231

[CR53] Public Health England. (2021). *Gambling-related harms evidence review: summary.* https://www.gov.uk/government/publications/gambling-related-harms-evidence-review/gambling-related-harms-evidence-review-summary

[CR54] Reilly, C., & Smith, N. (2013). The evolving definition of pathological gambling in the DSM-5. *National Center for Responsible Gaming,**1*, 1–6.

[CR55] Raymond, J. E., Shapiro, K. L., & Arnell, K. M. (1992). Temporary suppression of visual processing in an RSVP task: An attentional blink? *Journal of Experimental Psychology: Human Perception and Performance,**18*(3), 849–860. 10.1037/0096-1523.18.3.849 1500880 10.1037/0096-1523.18.3.849

[CR56] Rinck, M., & Becker, E. S. (2007). Approach and avoidance in fear of spiders. *Journal of Behavior Therapy and Experimental Psychiatry,**38*(2), 105–120. 10.1016/j.jbtep.2006.10.001 17126289 10.1016/j.jbtep.2006.10.001

[CR57] Robinson, T. E., & Berridge, K. C. (1993). The neural basis of drug craving: An incentive-sensitization theory of addiction. *Brain Research Reviews,**18*(3), 247–291. 8401595 10.1016/0165-0173(93)90013-P

[CR58] Ryan, R. (2016). Cochrane Consumers and Communication Review Group. ‘Heterogeneity and subgroup analyses in Cochrane Consumers and Communication Group reviews: planning the analysis at protocol stage. http://cccrg.cochrane.org, December 2016. Retrieved February 17 2023.

[CR60] Scottish Intercollegiate Guidelines Network (2012) *SIGN Methodology Checklist 4: Case-control studies*. https://www.sign.ac.uk/what-we-do/methodology/checklists/

[CR61] Shafran, R., Lee, M., Cooper, Z., Palmer, R. L., & Fairburn, C. G. (2007). Attentional bias in eating disorders. *International Journal of Eating Disorders,**40*, 369–380. 10.1002/eat.20375 17330290 10.1002/eat.20375 PMC2798076

[CR62] Sippel, L. M., Taverna, E., & Marshall, A. D. (2022). In vivo defensive behaviors, fear, and attention bias to physical and negative evaluation threats. *Behaviour Research and Therapy,**154*, 104108. 10.1016/j.brat.2022.104108 35596972 10.1016/j.brat.2022.104108

[CR63] Skinner, I. W., Hübscher, M., Moseley, G. L., Lee, H., Wand, B. M., Traeger, A. C., Gustin, S. M., & McAuley, J. H. (2018). The reliability of eyetracking to assess attentional bias to threatening words in healthy individuals. *Behavior Research Methods,**50*, 1778–1792. 10.3758/s13428-017-0946-y 28812285 10.3758/s13428-017-0946-y

[CR65] Sweller, J., van Merrienboer, J. J. G., & Paas, F. G. W. C. (1998). Cognitive architecture and instructional design. *Educational Psychology Review,**10*, 251–296. 10.1023/A:1022193728205 10.1023/A:1022193728205

[CR67] Tiffany, S. T. (1990). A cognitive model of drug urges and drug-use behavior: Role of automatic and nonautomatic processes. *Psychological Review,**97*(2), 147–168. 10.1037/0033-295x.97.2.147Z 2186423 10.1037/0033-295x.97.2.147Z

[CR68] Torday, J. S., & Baluška, F. (2019). Why control an experiment? From empiricism, via consciousness, toward implicate order. *EMBO Reports,**20*(10), e49110. 10.15252/embr.201949110 31482636 10.15252/embr.201949110 PMC6776925

[CR69] Vizcaino, E. V., Valladolid, G. R., Ponce, G., Moratti, S. S., Arriero, M. J., Fernandez, P., & Blanco, C. (2012). P-103-Dot probe task to assess attentional bias in pathological gamblers (PG). *European Psychiatry,**27*(S1), 1–1.22153731 10.1016/S0924-9338(12)74270-0

[CR70] Winters, K. C., Stinchfield, R. D., & Fulkerson, J. (1993). Toward the development of an adolescent gambling problem severity scale. *Journal of Gambling Studies,**9*(1), 63–84. 10.1007/BF01019925 10.1007/BF01019925

[CR71] Wittekind, C. E., Bierbrodt, J., Lüdecke, D., Feist, A., Hand, I., & Moritz, S. (2019). Cognitive bias modification in problem and pathological gambling using a web-based approach-avoidance task: A pilot trial. *Psychiatry Research,**272*, 171–181. 10.1016/j.psychres.2018.12.075 30583260 10.1016/j.psychres.2018.12.075

[CR72] Wölfling, K., Mörsen, C. P., Duven, E., Albrecht, U., Grüsser, S. M., & Flor, H. (2011). To gamble or not to gamble: At risk for craving and relapse—learned motivated attention in pathological gambling. *Biological Psychology*. 10.1016/j.biopsycho.2011.03.010 21453747 10.1016/j.biopsycho.2011.03.010

[CR73] Zack, M., & Poulos, C. X. (2004). Amphetamine primes motivation to gamble and gambling-related semantic networks in problem gamblers. *Neuropsychopharmacology,**29*(1), 195–207. 10.1038/sj.npp.1300333 14571257 10.1038/sj.npp.1300333

[CR74] Zack, M., & Poulos, C. X. (2007). A D2 antagonist enhances the rewarding and priming effects of a gambling episode in pathological gamblers. *Neuropsychopharmacology,**32*(8), 1678–1686. 10.1038/sj.npp.1301295 17203013 10.1038/sj.npp.1301295

